# Genetic Variation among the Partial Gene Sequences of the Ribosomal Protein Large-Two, the Internal Transcribed Spacer, and the Small Ribosomal Subunit of *Blastocystis* sp. from Human Fecal Samples

**DOI:** 10.3390/microorganisms12061152

**Published:** 2024-06-05

**Authors:** Guiehdani Villalobos, Eduardo Lopez-Escamilla, Angelica Olivo-Diaz, Mirza Romero-Valdovinos, Arony Martinez, Pablo Maravilla, Fernando Martinez-Hernandez

**Affiliations:** 1 Departamento de Produccion Agricola y Animal, Universidad Autonoma Metropolitana, Mexico City 04960, Mexico; guiehda@yahoo.com.mx; 2 Departamento de Biologia Molecular e Histocompatibilidad, Hospital General “Dr. Manuel Gea Gonzalez”, Mexico City 14080, Mexico; eduar1escamilla@gmail.com (E.L.-E.); aolivod@yahoo.com (A.O.-D.); 3 Laboratorio de Patogenos Emergentes, Departamento de Biologia Molecular e Histocompatibilidad, Hospital General “Dr. Manuel Gea Gonzalez”, Mexico City 14080, Mexico; mirzagrv@yahoo.com; 4 Departamento de Ecologia de Agentes Patogenos, Hospital General “Dr. Manuel Gea Gonzalez”, Mexico City 14080, Mexico; arony@live.com.mx

**Keywords:** *Blastocystis* sp., internal transcribed spacer region (ITS), small subunit ribosomal DNA (SSUrDNA), ribosomal protein large two gene (*rpl2*), subtypes (STs)

## Abstract

In the present study, we compared the genetic variability of fragments from the internal transcribed spacer region (ITS) and the small subunit ribosomal DNA (SSUrDNA) as nuclear markers, in contrast with the ribosomal protein large two (*rpl2*) *loci*, placed in the mitochondrion-related organelles (MROs) within and among human fecal samples with *Blastocystis*. Samples were analyzed using polymerase chain reaction (PCR)-sequencing, phylogenies, and genetics of population structure analyses were performed. In total, 96 sequences were analyzed, i.e., 33 of SSUrDNA, 35 of *rpl2*, and 28 of ITS. Only three subtypes (STs) were identified, i.e., ST1 (11.4%), ST2 (28.6%), and ST3 (60%); in all cases, kappa indexes were 1, meaning a perfect agreement among ST assignations. The topologies of phylogenetic inferences were similar among them, clustering to each ST in its specific cluster; discrepancies between phylogeny and assignment of STs were not observed. The STRUCTURE v2.3.4 software assigned three subpopulations corresponding to the STs 1–3, respectively. The population indices were consistent with those previously reported by other groups. Our results suggest the potential use of the ITS and *rpl2* genes as molecular markers for *Blastocystis* subtyping as an alternative approach for the study of the genetic diversity observed within and between human isolates of this microorganism.

## 1. Introduction

*Blastocystis* is a non-flagellated, anaerobic, unicellular stramenopile that commonly inhabits the intestinal tract of humans and animals and is widespread throughout the world. Despite more than 1 billion carriers worldwide, the public health significance remains unknown [1,2]; the blastocystosis prevalence rates vary between countries and within communities, being higher in developing countries than in industrialized countries [3].

This parasite exhibits extensive morphological and genetic variation [4]. Morphological forms include vacuolar, granular, amoeboid, and cyst, each one of which also shows heterogeneity in size [5,6]. Although the isolates from different hosts might appear morphologically similar, *Blastocystis* from mammals and birds exhibits a high genetic polymorphism comprised of up to 44 ribosomal lineages known as subtypes (STs) [7,8]. These have been described by analyzing the small subunit ribosomal DNA (SSUrDNA), and some STs are as divergent as species or even genera [2]. In humans, at least 14 STs have been identified, ST1 to ST10, ST12, ST14, ST16, and ST23, with STs 1 to 4 being the most common, while the rest are infrequent or rare [9,10,11,12,13]. The remarkable genetic heterogeneity of *Blastocystis* has been studied by polymerase chain reaction (PCR) with SSUrDNA genotyping [2]. Each one of the genetic tools used has strengthened the findings of the incredible genetic diversity that this parasite presents. However, so far, it has not been possible to establish a clear association between the genetic diversity of subtypes and characteristics such as host specificity, epidemiology, and potential clinical relevance. Analysis of diverse gene segments or the new sequencing technologies allows a better overview of the differences between each group, helping to discriminate subtypes, chimeras, or coinfections. For instance, the internal transcribed spacer region ITS1+5.8S+ITS2 (ITS) was described to be more polymorphic than SSUrDNA, offering reliable results on the genetic variability within populations and, therefore, can be used as a genetic marker of *Blastocystis*; it also allows distinguishing between different subtypes using only one PCR amplification applied directly to fecal samples [14]. However, the study of subtypes has been limited to the study of a few genes, mainly of nuclear origin. The capacity of other markers of genetic diversity and their impact on the biological study of the parasite, such as those mitochondrion-related organelles (MROs), has been little explored. *Blastocystis* MROs have characteristics of typical mitochondria, including Complexes I and II from the electron transport chain, mitochondrial DNA, Fe-S cluster assembly, and amino acid metabolism, as well as common proteins identified in obligate anaerobes, including Fe-hydrogenase, pyruvate metabolism, and alternative oxidase [15]. Eight *Blastocystis* mitochondrial genomes (STs 1–4, 6–9) are available, although not all are assembled [16,17,18]. Twenty-seven protein-coding genes (including *rpl2*), ribosomal RNA genes, and 16 transfer RNA genes had been described; interestingly, phylogenetic analysis showed an identical topology between the MRO and SSUrDNA genomic sequences for the three main clades grouping *Blastocystis* STs. [18]. Since some differences between nuclear genes and isolates within the same *Blastocystis* STs have been widely documented, and each gene tells a different evolutionary story, it is interesting to explore in parallel the genetic variation at different nuclear and MRO *loci* among *Blastocystis* isolates.

The objective of the present study was to compare the genetic variability of two nuclear (ITS and SSUrDNA) and one MRO (*rpl2*) *locus* among isolates of *Blastocystis* ST1-3 from human carriers.

## 2. Materials and Methods

### 2.1. DNA Extraction and PCR Development

Thirty-six frozen fecal samples stored at −70 °C in 70% ethanol from gastrointestinal symptomatic Mexican *Blastocystis* carriers that were collected but not analyzed for previous studies and whose participants gave written informed consent [14,19] were recovered and processed for the present study. DNA was extracted from approximately 250 mg of feces using a ZR Fecal DNA MiniPrep kit (Zymoresearch, CA, USA) according to the manufacturer’s protocol. Three different DNA segments were amplified and sequenced: (1) a partial SSUrDNA fragment (~500 bp) was obtained using the primers and PCR conditions reported by Santin et al. [20]; (2) for ITS region (from ~530 to 620 bp, depending of *Blastocystis* subtype) the set of primers and PCR conditions for amplifying were based on reported by Villalobos et al. [14]; (3) finally, for *rpl2 loci* (~600 bp) primers were designed based on the alignment from STs sequences available of *Blastocystis* deposited GenBank; briefly, the MROs genome full sequences of ST1, ST4, and ST7 were aligned using Clustal W [21]; the region that includes almost the entire gene was selected and amplified using *rpl2*_F (5-AAG TGG TAG AAA TTT TCG WGG-3′) and *rpl2*_R (5′-GTA ATT AAA CCC CAA GGW GT-3′) primers. PCR amplifications were carried out in a final volume of 50 µL containing 50 pMol of each primer, 1X PCR buffer (8 mM Tris-HCl, pH 8, 20 mM KCl), 2.5 mM MgCl2, 0.5 mM dNTPs, 2 U Taq DNA Polymerase (Invitrogen, Carlsbad, CA, USA), and 100 ng of DNA. PCR products were analyzed with 1.2% agarose gel electrophoresis, visualized by ethidium bromide staining (0.5 µg/mL), and purified with an AxyPrep PCR clean-up kit (Axigen Biosciences, Union City, CA, USA). A commercial supplier sequenced all purified products in both directions.

All sequences were subjected to a BLAST search for genetic identification and submitted to the GenBank database to obtain access numbers (PP592306-33, PP597305-39, and PP587181-PP587213); then, multiple alignments were performed using the CLUSTAL W (accessed on 6 February 2024) [21] and MUSCLE v3.8.31 [22] tools in the MEGA 10.1.8 program [23].

### 2.2. Phylogenetic Analysis

The phylogenetic trees were built using the molecular evolution model, the general time-reversible model with a gamma distribution (GTR+G) for ITS and *rpl2* sequences, and the general time-reversible model with a gamma distribution and invariant sites (GTR+G+I) for SSUrDNA. Bayesian algorithm reconstructions were performed using the MrBayes 3.2.7a software [24]; the analysis was performed for two million generations with sampling trees every 100 generations. Trees with scores below the burn-in phase were discarded, and the remainder were collected and used to build majority consensus trees.

A median-joining network analysis was performed using NETWORK 4.6 [25], and haplotype networks were established under default settings and assumptions. For all analysis, the following sequences of *Blastocystis* available in GenBank were used as controls of subtypes, i.e., ST1: HQ641595-6 for SSUrDNA, AY125913-9, KM213469-72, KM213475, and KM213477 for ITS, EF494740 for *rpl2*; ST2: HQ641602, HQ641654, and JX305874-5 for SSUrDNA, KM213492-5 for ITS; ST3: JX305879, HQ641613, JX305880, JX305883, and HQ641611 for SSUrDNA, KM213505-8 for ITS, CU914152, EF494739, HQ909887, JZRK01000047, HQ909886, and HQ909888 for *rpl2*.

### 2.3. Genetics of Populations Analysis

The STRUCTURE analysis [26] (https://web.stanford.edu/group/pritchardlab/structure_software/release_versions/v2.3.4/html/structure.html, accessed on 5 March 2024) was carried out to determine the most probable number of clusters across all *Blastocystis* samples with each gene. The value of K, or a theoretical number of the populations independent of the subtypes, was obtained using the predetermined values of the software: correlated allele frequencies and admixture. A single run of the algorithm started with an initial random association of alleles in K clusters and was established with ten independent replicates for each value of K from 1 to 10 and with a length of burning period of 10,000 and a number of MCMC reps after burning of 100,000. The appropriate number of clusters was determined by calculating the delta K value [27]. A second run was performed using the delta K value assigned in the program (K = 3). This new analysis was run with a burning period length of 50,000 and a number of MCMC reps after burning of 200,000. The probabilities of group membership were as follows: 0.997–0.999 for K1, 0.999 for K2, and 0.993–0.999 for K3 for 18S; 0.996–0.999 for K1, 0.985-0.999 for K2, and 0.979–0.999 for K3 for ITS; and 0.994–0.999 for K1, 0.739–0.999 for K2, and 0.988–0.999 for K3 for rpl2.

The genetic diversity indexes among subtypes and *locus* were performed using DnaSPv5 [28]; specifically, haplotype diversity (Hd), nucleotide diversity (π, average number of nucleotide differences among all possible pairs of sequences in the sample), haplotype polymorphism (θ, proportion of nucleotide sites that can be predicted to be polymorphic from this region of the genome), and Tajima’s D (the tests indicate a balancing selection with a positive value and purifying selection or recent expansion process if the value is negative) were calculated, according to Hedrick interpretation [29].

### 2.4. Statistical Analysis

To assess the agreement among positivity for *loci* analyzed, Cohen’s kappa index was estimated using the IBM SPSS statistics for Windows. Version 27 (Armonk, NY, USA: IBM Corp.).

## 3. Results

### 3.1. Blastocystis Subtyping

In total, ninety-six sequences were analyzed: 33 with SSUrDNA marker, 35 with *rpl2*, and 28 with ITS. Table 1 shows the subtyping obtained for each of the samples for the three different *loci* studied. Although subtyping was performed in most samples by sequencing the three *loci*, in 9 cases, sequencing of one *locus* failed, and in only one sample was subtyped by analysis of one *locus*. Only three subtypes were identified: ST1 (11.4%), ST2 (28.6%), and ST3 (60%). In all cases, the kappa values obtained were 1 (Table 2), meaning a perfect agreement [30].

### 3.2. Phylogenetic Inferences

**Figure 1:**
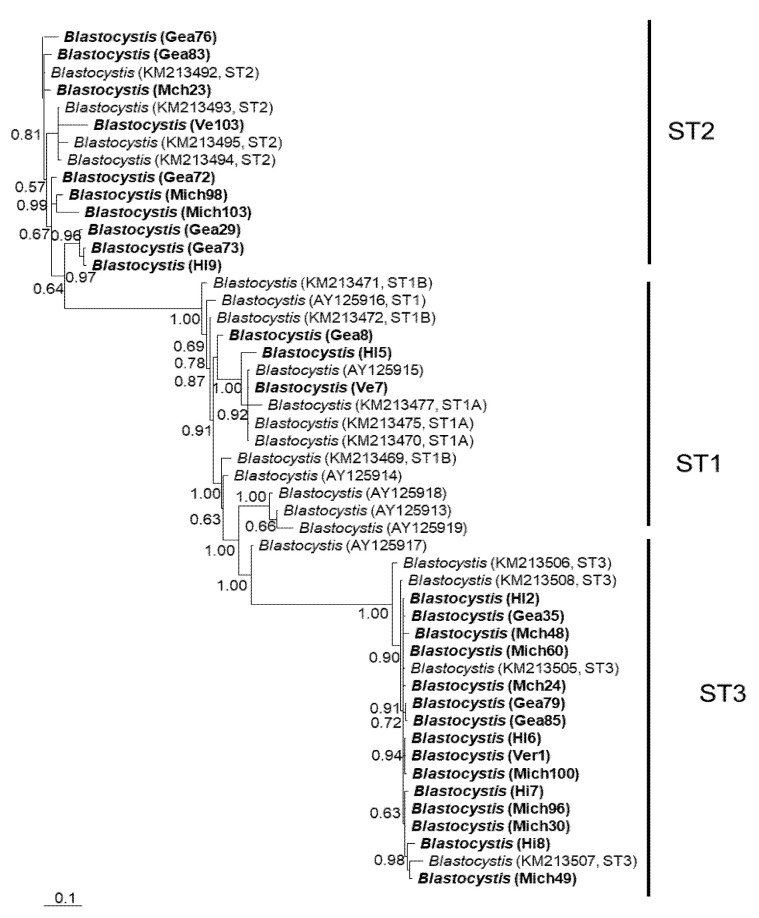
Bayesian phylogenetic tree based on ITS1+5.8S+ITS2 sequences of *Blastocystis*. The numbers at the nodes indicate Bayesian posterior probabilities. The sequences obtained in this study are shown in bold.

**Figure 2:**
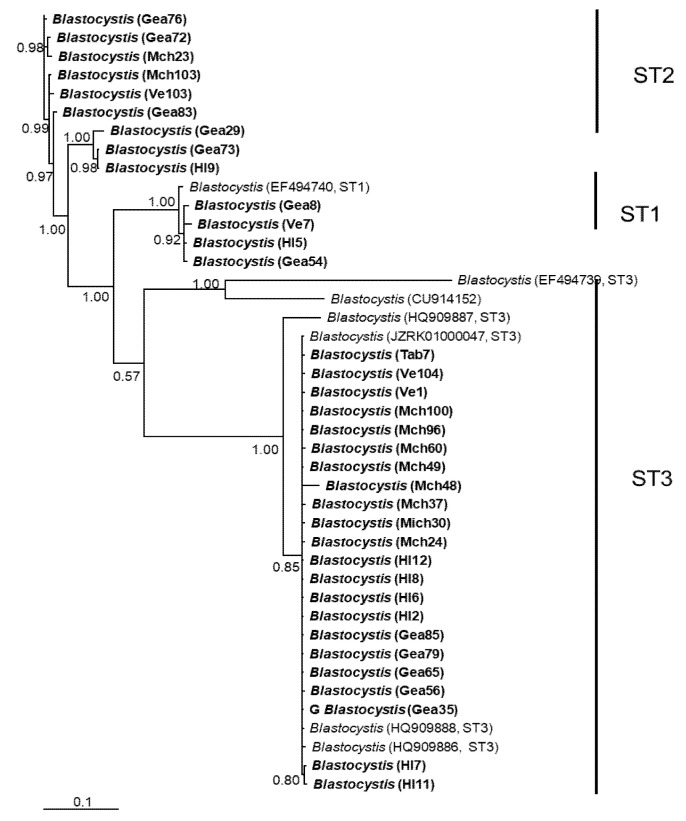
Bayesian phylogenetic tree based on *rpl2* sequences of *Blastocystis*. The numbers at the nodes indicate Bayesian posterior probabilities. The sequences obtained in this study are shown in bold.

**Figure 3:**
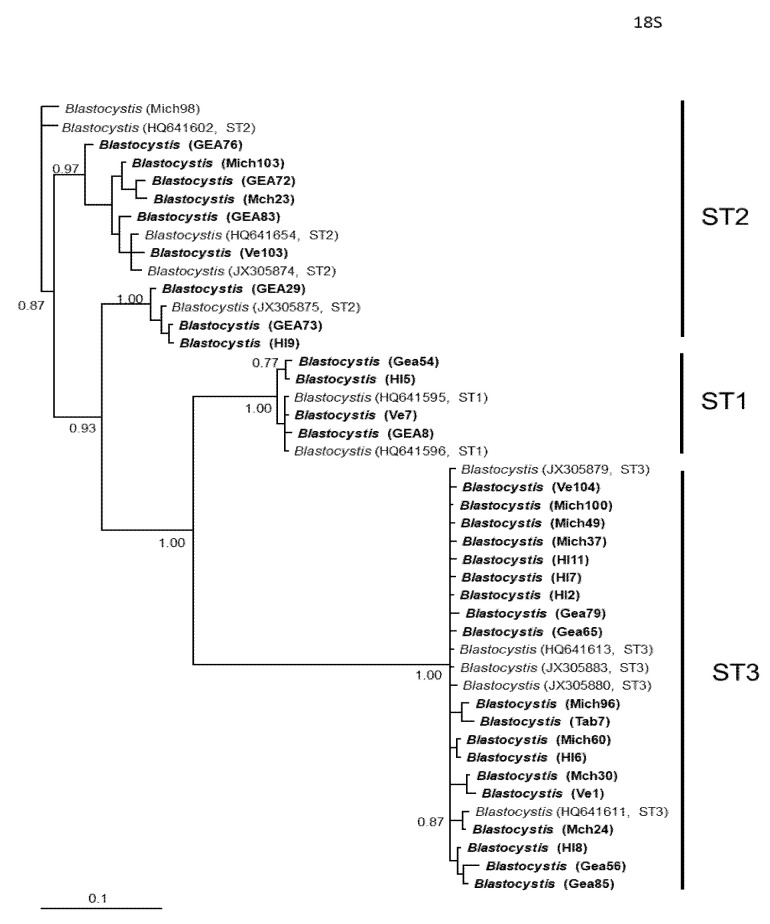
Bayesian phylogenetic tree based on SSUrDNA sequences of *Blastocystis*. The numbers at the nodes indicate Bayesian posterior probabilities. The sequences obtained in this study are shown in bold.

In general, the topology of the phylogenetic trees for the three *loci* was similar, supported by high posterior probability values (Figure 1, Figure 2 and Figure 3). The most homogeneous clusters were observed for ST1 and 3; particularly, ITS ST1 sequences were grouped only in subgroup A (Figure 1; ST1A) cluster, in accordance with what was previously documented for this marker [14]. Due to the lack of available sequences for ITS and *rlp2* for the different *Blastocystis* STs, only trees with STs 1–3 are shown here; however, for SSUrDNA, a phylogenetic tree was built with all recognized subtypes, and its topology was concordant with the tree for STs 1–3 (Figure 3).

**Figure 4:**
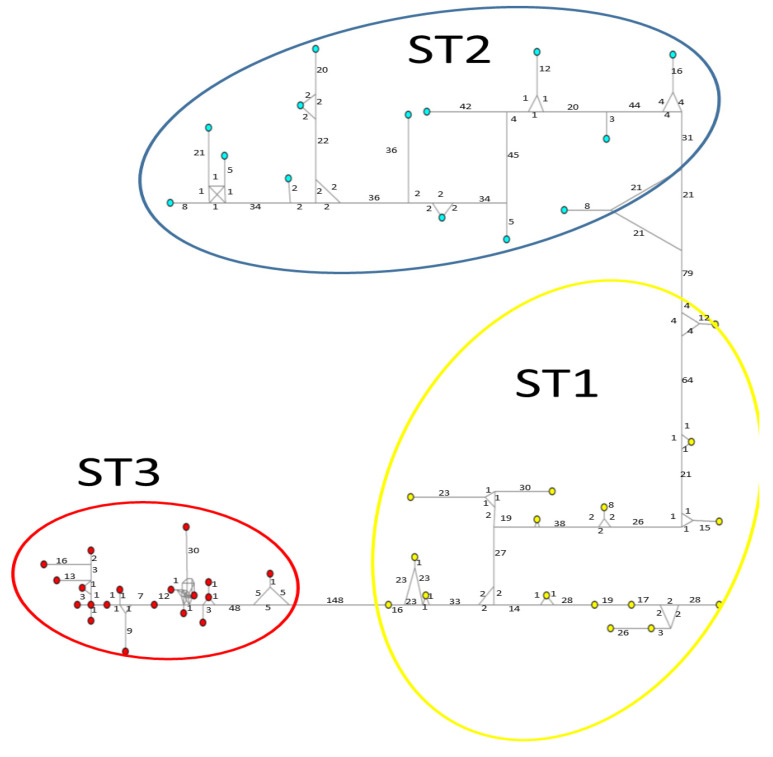
A median-joining haplotype network tree that was generated using ITS1+5.8S+ITS2 sequences of *Blastocystis*; the numbers on branches refer to mutational changes. Haplotypes for *Blastocystis* ST1 are shown in yellow, ST2 in blue, and ST3 in red. Numbers in branches are mutational changes; sizes of circles are proportional to haplotype frequencies.

**Figure 5:**
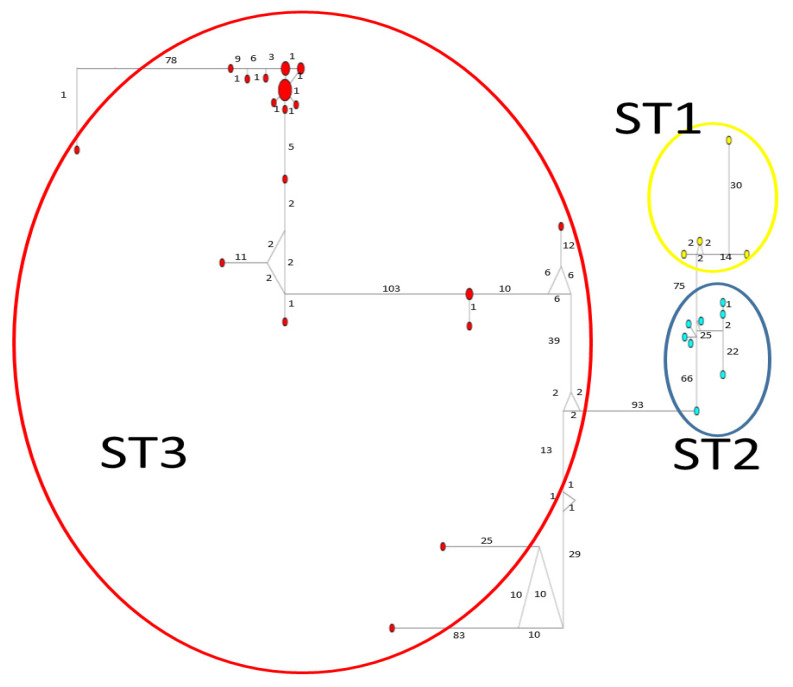
A median-joining haplotype network tree that was generated using *rpl2* sequences of *Blastocystis*; the numbers on branches refer to mutational changes. Haplotypes for *Blastocystis* ST1 are shown in yellow, ST2 in blue, and ST3 in red. Numbers in branches are mutational changes; sizes of circles are proportional to haplotype frequencies.

**Figure 6:**
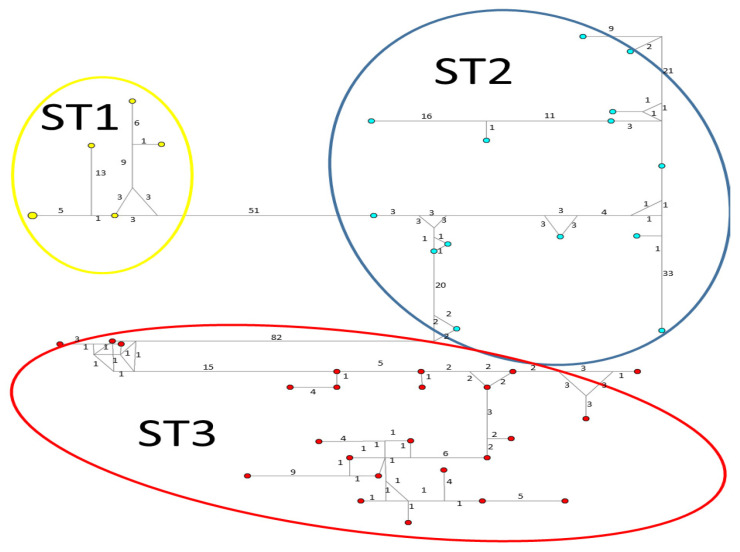
A median-joining haplotype network tree that was generated using SSUrDNA sequences of *Blastocystis*; the numbers on branches refer to mutational changes. Haplotypes for *Blastocystis* ST1 are shown in yellow, ST2 in blue, and ST3 in red. Numbers in branches are mutational changes; sizes of circles are proportional to haplotype frequencies.

Considerable mutation steps within STs were observed in the haplotype network (Figure 4, Figure 5 and Figure 6) for all STs. In *rpl2*, contrary to the phylogenetic tree, the greatest variation observed was in ST3 (Figure 5), where the mutation steps were up 100, even greater than those that separate the subtypes. In ITS (Figure 4), the network showed variation in ST1, while the other subtypes were consistent with their cluster, similar to that exhibited by SSUrDNA (Figure 6).

### 3.3. Genetics of Population Structure

**Figure 7:**
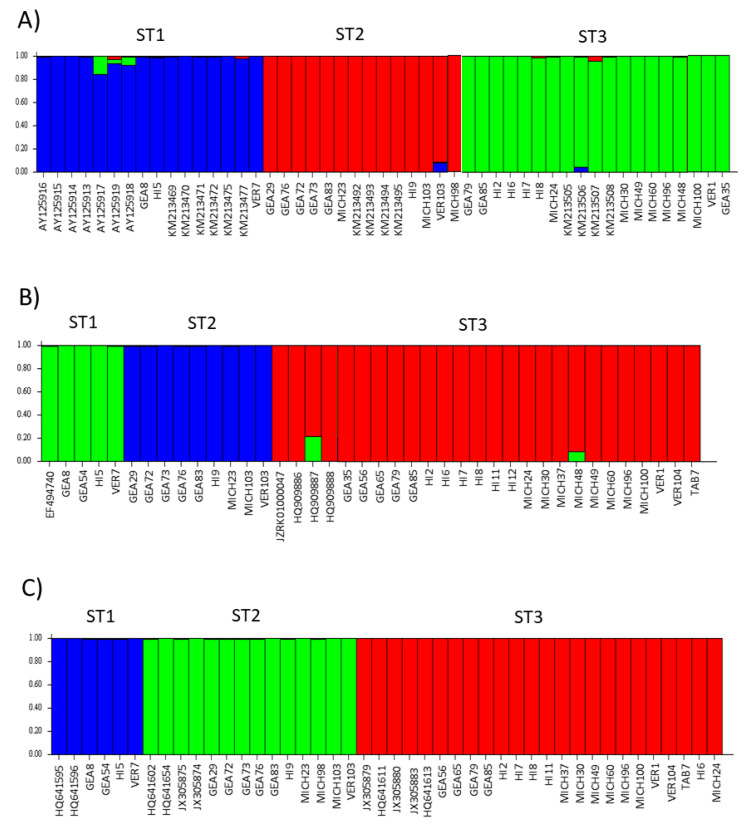
Three genetic structure clusters (indicated by colors) using ITS in (**A**), *rpl2* in (**B**), and SSUrDNA in (**C**) sequences of *Blastocystis*, according to the Bayesian MCMC simulation. Each sample is represented by a thin vertical line divided into colored segments that indicate one of the K3 genetic clusters.

The STRUCTURE software assigned the samples studied here to three subpopulations, and these subpopulations corresponded to STs 1–3, respectively (Figure 7). There were no mixed samples that could not be assigned to any of the three existing subpopulations. Interestingly, the bar plot for ITS showed more diversity of colors in each subpopulation, i.e., few samples with more than one color were observed; in contrast, the *rpl2* and SSUrRNA sequences were completely homogeneous. The population indices were generally concordant among the three *loci* analyzed (Table 3). Comparisons between STs showed that ST3 exhibited the lowest π values with all three *loci*. The only significant value in Tajima’s D was for ST3 with *rpl2* (−2.1949).

## 4. Discussion

*Blastocystis* exhibits large genetic diversity, presenting 44 STs based on SSUrDNA analysis [8], and it is precisely this great genetic diversity in this microorganism that has motivated numerous studies. Since knowledge of the genetic structure of parasites can be useful for its control, genetic variation within and between populations determines the future evolutionary changes, as well as the processes of differentiation and genetic speciation [14,31]; therefore, further studies on the genetics of populations of *Blastocystis* could help clarify the biological processes mentioned above.

In the present study, the genetic variabilities of two nuclear *loci* (SSUrDNA and ITS) and one mitochondrial *locus* (*rpl2*) of *Blastocystis* isolates were compared. In general, clear amplicons and sequences were yielded during the amplification of the three *loci*; however, for the SSUrDNA *locus*, eight samples could not be amplified (22%); for the ITS, three samples could not be amplified (12%), and for *rpl2*, only one sample could not be amplified (3%). These differences in the detection percentage could be due to failures during the DNA extraction, considering that unpreserved stool samples were stored frozen at −70 °C for ten years and that false negatives may occur if the analysis is performed directly by PCR-stool instead of PCR-culture [32]. In addition, it has been clearly documented that commercial stool DNA extraction systems have different detection rates [33]; in the present study, the commercial system used (Zymoresearch, CA, USA) previously showed a higher success rate in amplifying diagnostic PCR for *Blastocystis* [33].

Regarding the three STs identified (STs 1–3), these have been identified as the most frequent in Mexico, in both urban and rural populations [14,34,35,36,37,38]. Phylogenetic inferences and haplotype network trees unequivocally grouped the samples into clusters corresponding to STs 1–3. Since no discrepancies in the location of STs were observed in the distribution and topology within the phylogenetic and haplotype trees, it makes sense to rule out possible coinfections or mixtures of STs, as suggested by Poirier et al. [39], when during the analysis of a single copy *Blastocystis* MRO *locus*, discrepancies in phylogeny and ST assignment were observed and the presence of a mixture of STs were confirmed by cloning. Although a wide diversity of genetic markers can be designed from DNA regions of both the nuclear and mitochondrial genomes, the resolution and usefulness of each depends largely on the degree of variation in the marker sequence [40]. Since mitochondrial genes evolve at higher rates than nuclear genes, a greater degree of nucleotide variation can be detected, which makes it a potentially useful source of genetic markers for studies of closely related taxa or to resolve lower taxonomic levels for organisms [41,42]. Therefore, the analysis of mitochondrial markers of *Blastocystis* arises as a complementary approach to support the potential implications of variability in this microorganism. The *rpl2* gene is located in a conservative cluster of ribosomal protein genes, *rpl2-rps19-rps3-rpl16*, in the MRO genome; the size of the *rpl2* gene is approximately 753 bp, and its variation can range from 12 to 20% of informative sites, making it a good candidate for the genetic study of the parasite [16].

The ST3 had the lowest values of genetic variability compared to the other STs. This is in accordance with other previous studies [35,36]; also, the only statistically significant negative value obtained in Tajima’s D was for *rpl2* in ST3; this negative value indicates a population expansion process with a high frequency of circulating rare alleles [43]; thus, this is in concordance with other reports in which this ST was more genetically diversified than other STs [36,44]. Interestingly, nuclear markers “nuclear genes are less affected by deleterious mutations associated with asexual reproduction” [45], where we expect ITS to be less diverse than mitochondrial; however, together with SSUrDNA, both present higher values of π with respect to *rpl2* (0.146 and 0.138, respectively, versus 0.098).

The STRUCTURE software is a common tool widely used for population analysis because it supports researchers to assess patterns of genetic structure in a set of samples, assigning individuals to subpopulations based on analysis of likelihoods [46]. There are many examples of articles about the usefulness of the STRUCTURE program to establish new variants in parasites such as *Toxoplasma gondii* [47,48] and *Plasmodium falciparum* [49]. Here, the STRUCTURE bar plots for the three *loci* used clearly revealed the presence of three subpopulations corresponding to STs 1–3, respectively.

## 5. Conclusions

We described the potential use of ITS and *rpl2* genes as molecular markers for *Blastocystis* subtyping. However, the main goal was to provide tools that contribute to supporting the genetic diversity observed within and among isolates; thus, as an approach, studying the *loci* separately opens the possibility of future analyses focused on intrasubtype variability. Certainly, only fragments of the SSUrDNA, ITS, and *rpl2* genes were explored, so care must be taken in the scope of the results obtained; however, our results are consistent with other reports of published evidence on metabolic and genetic differences of STs 1–3, showing that these STs could be assigned as different cryptic species. Therefore, although the term subtype (understood as DNA sequences that group together as a discrete clade within the range of diversity of a defined species) remains accepted, it should be considered a future consensus on the evaluation of species in *Blastocystis* STs 1–3 [50,51,52].

## Figures and Tables

**Table 1:** Samples (GenBank accession number) and subtyping by ITS, *rpl2,* and SSUrDNA analysis.

No.	Sample	Subtype (*GenBank Accession Number*)		
		ITS	SSUrDNA	*rpl2*
1	GEA 8	**1** (*PP592306*)	**1** (*PP587181*)	**1** (*PP597305*)
2	GEA 29	**2** (*PP592329*)	**2** (*PP587182*)	**2** (*PP597309*)
3	GEA 35	**3** (*PP592307*)	ND	**3** (*PP597318*)
4	GEA 54	ND *	**1** (*PP587183*)	**1** (*PP597308*)
5	GEA 56	ND	**3** (*PP587184*)	**3** (*PP597319*)
6	GEA 65	ND	**3** (*PP587185*)	**3** (*PP597320*)
7	GEA 72	**2** (*PP592308*)	**2** (*PP587186*)	**2** (*PP597310*)
8	GEA 73	**2** (*PP592309*)	**2** (*PP587187*)	**2** (*PP597311*)
9	GEA 76	**2** (*PP592310*)	**2** (*PP587188*)	**2** (*PP597312*)
10	GEA 79	**3** (*PP592311*)	**3** (*PP587189*)	**3** (*PP597321*)
11	GEA 83	**2** (*PP592312*)	**2** (*PP587190*)	**2** (*PP597313*)
12	GEA 85	**3** (*PP592313*)	**3** (*PP587191*)	**3** (*PP597322*)
13	HI2	**3** (*PP592314*)	**3** (*PP587192*)	**3** (*PP597323*)
14	HI5	**1** (*PP592315*)	**1** (*PP587193*)	**1** (*PP597306*)
15	HI6	**3** (*PP592316*)	**3** (*PP587194*)	**3** (*PP597326*)
16	HI7	**3** (*PP592317*)	**3** (*PP587195*)	**3** (*PP597325*)
17	HI8	**3** (*PP592318*)	**3** (*PP587196*)	**3** (*PP597326*)
18	HI9	**2** (*PP592319*)	**2** (*PP587197*)	**2** (*PP597314*)
19	HI11	ND	**3** (*PP587198*)	**3** (*PP597327*)
20	HI 12	ND	ND	**3** (*PP597328*)
21	Mich 23	**2** (*PP592320*)	**2** (*PP587199*)	**2** (*PP597315*)
22	Mich 24	**3** (*PP592321*)	**3** (*PP587200*)	**3** (*PP597329*)
23	Mich 30	**3** (*PP592322*)	**3** (*PP587201*)	**3** (*PP597330*)
24	Mich 37	ND	**3** (*PP587202*)	**3** (*PP597331*)
25	Mich 48	**3** (*PP592323*)	ND	**3** (*PP597332*)
26	Mich 49	**3** (*PP592324*)	**3** (*PP587203*)	**3** (*PP597333*)
27	Mich 60	**3** (*PP592325*)	**3** (*PP587204*)	**3** (*PP597334*)
28	Mich 96	**3** (*PP592326*)	**3** (*PP587205*)	**3** (*PP597335*)
29	Mich 98	**2** (*PP592327*)	**2** (*PP587206*)	ND
30	Mich 100	**3** (*PP592328*)	**3** (*PP587207*)	**3** (*PP597336*)
31	Mich 103	**2** (*PP592330*)	**2** (*PP587208*)	**2** (*PP597316*)
32	Tab 7	ND	**3** (*PP587209*)	**3** (*PP597337*)
33	Ve 1	**3** (*PP592331*)	**3** (*PP587210*)	**3** (*PP597338)*
34	Ve 7	**1** (*PP592332*)	**1** (*PP587211*)	**1** (*PP597307*)
35	Ve 103	**2** (*PP592333*)	**2** (*PP587212*)	**2** (*PP597317*)
36	Ve 104	ND	**3** (*PP587213*)	**3** (*PP597339*)
Total		28	33	35

* ND: not determined.

**Table 2:** Cohen’s kappa coefficient (k) results for the comparison of subtyping between *loci*.

Comparison	k Value	*p* Value
ITS vs. *rpl2*	1.0	1.386 × 10^−10^
ITS vs. SSUrDNA	1.0	1.966 × 10^−10^
*rpl2* vs. SSUrDNA	1.0	3.371 × 10^−12^

**Table 3:** Genetic polymorphism indexes between different *Blastocystis* sequences.

*Loci*/ST	n	h	Hd	π	Θ	Tajima’s D	*p* Value
ITS	28	16	0.823	0.14663	0.10865		
ST1	3	3	1	0.05394	0.05256	ND	
ST2	10	9	0.978	0.05889	0.0707	−1.25023	*p* > 0.05
ST3	15	8	0.829	0.00608	0.00995	−1.7122	*p* > 0.05
SSUrDNA	33	20	0.934	0.13806	0.07457		
ST1	4	4	1	0.00598	0.00559	0.6501	*p* > 0.05
ST2	10	8	0.933	0.05737	0.03928	0.50505	*p* > 0.05
ST3	19	10	0.877	0.00458	0.00511	−0.96514	*p* > 0.05
*rpl2*	35	7	0.644	0.09839	0.05845		
ST1	4	4	1	0.00681	0.00743	−0.81734	*p* > 0.05
ST2	9	3	0.556	0.01222	11,107	0.38489	*p* > 0.05
ST3	22	8	0.545	0.00159	0.00481	−2.1949	*p* < 0.05

n, number of samples; h, haplotypes; Hd, haplotype diversity; π, nucleotide diversity; θ, haplotype polymorphism; ND, not determined.

## Data Availability

All relevant data are within the article. The sequence data were submitted to the GenBank database under the accession numbers PP592306-33, PP597305-39, and PP587181-PP587213.
